# Initial Presentation of OCD and Psychosis in an Adolescent during the COVID-19 Pandemic

**DOI:** 10.1155/2022/2501926

**Published:** 2022-04-15

**Authors:** Sahana Nazeer, Abhishek Reddy

**Affiliations:** ^1^Virginia Tech Carilion School of Medicine, 2 Riverside Circle, Roanoke VA 24016, USA; ^2^Department of Psychiatry and Behavioral Medicine, 2017 South Jefferson Street, Roanoke VA 24016, USA

## Abstract

The COVID-19 pandemic is unparalleled in recent history when accounting for the true disease burden and dramatic impact on physical and mental health. Due to its infectious pathology, COVID-19 presents with a variety of symptoms including neuropsychiatric complications. Moreover, factors such as quarantine, social isolation, and fear of illness have negatively impacted the health of non-COVID-19 patients. There has been significant literature reporting new-onset psychiatric illness in all global populations including those without history of psychiatric illness. This report discusses an adolescent male without prior psychiatric history presenting with new onset symptoms of obsessive-compulsive disorder and psychosis in the context of COVID-19. There are considerable reports describing new-onset obsessive-compulsive disorder, albeit conflicting in terms of prevalence and exacerbations in the setting of COVID-19 in both adult and adolescent populations but limited reports of new-onset psychosis in those same populations and setting.

## 1. Introduction

The coronavirus 2019 (COVID-19) emerged in Wuhan, China, and as of December 2021, there have been more than 270 million confirmed cases, including 5.3 million deaths [1]. It is widely accepted that these measurements are underestimations of the true disease burden. The severe acute respiratory syndrome 2 (SARS-CoV-2) infection causing COVID-19 typically presents with respiratory symptoms, but outcomes have ranged from asymptomatic or mild to rapidly fatal.

Due to its spike-like glycoproteins attaching to the widespread angiotensin-conversion enzyme 2 (ACE2), COVID-19 presents with a variety of symptoms although the pathophysiology of SARS-CoV-2's neuropsychiatric complications such as anxiety, psychosis, delirium, and depression are yet to be fully determined [2]. It is well supported that the COVID-19 pandemic has exacerbated psychiatric disorders in patients with psychiatric history via measures such as quarantine, social distancing, and particularly social isolation [3–5]. It has also been reported that neuroinvasive infections such as COVID-19 can increase the risk of developing psychotic disorders through inflammatory mechanisms especially during key stages of physiologic neurodevelopment [6].

Obsessive-compulsive disorder (OCD), a relapsing and remitting disorder impacting function, is defined by recurrent obsessions and compulsions, of which contamination and cleaning is common [7]. There was an increase in the severity of obsessions and compulsions from the beginning of the pandemic despite remission of OCD symptoms prior to the pandemic [8].

Psychosis is defined by impairment in reality testing, often including hallucinations without insight and/or delusions leading to functional impairment. It is also reported to onset from fear of COVID-19 in patients without prior psychiatric history [9–14], as exacerbate paranoia in patients with psychiatric history [15], after recovery from SARS-CoV-2 infection [16], or during a COVID-19 infection [17–21]. The onset and worsening of psychosis are associated with psychosocial factors such as increased stress from a pandemic, such as the increase in psychosis during the influenza epidemic [22, 23].

The exacerbation of mental health concerns, especially anxiety and depression, due to COVID-19 has also been consistently reported [4, 24, 25]. There are conflicting reports of the prevalence and onset of OCD in the context of COVID-19 as certain sources state a greater prevalence of OCD and worsening of existing OCD symptoms during the COVID-19 pandemic whereas others detail a lack of OCD symptom deterioration [26–28]. Moreover, there have been multiple case reports that describe the emergence of new-onset OCD in both adolescents and adults [29–31] as well as case reports delineating exacerbations of preexisting OCD during the COVID-19 pandemic [32–34].

This case report adds to the growing literature of new-onset OCD and the minimal literature of new-onset psychosis in adolescent patients due to COVID-19. We describe an adolescent male without previous psychiatric history who presented with newly onset obsessive-compulsive disorder and symptoms of psychosis triggered by the COVID-19 pandemic. We outline the patient's initial presentation, two psychiatric hospitalizations, outpatient follow-ups, and gradual improvement.

## 2. Case Report

A male teenager with no significant previous medical, surgical, or psychiatric history presented with gradually worsening isolation and concern about cleanliness for months since the initiation of remote learning secondary to the severe acute respiratory syndrome coronavirus 2 (SARS-CoV-2) pandemic. The patient had symptoms of repetitive and excessive cleaning of his home, lasting about half the day, except for his personal bedroom, which remained messy and even dirty. Due to his fear of contamination from the rest of the home, the patient did not wear clothes in other areas of his house except his bedroom and did not allow others into his bedroom. Prior to the pandemic, the patient received high grades and participated in extracurriculars. Due to the pandemic, he opted for virtual school and, consequently, struggled with his academics. There was no reported history of substance use at this time. The patient does not have a family history of psychiatric illness and did not have any concern with meeting his early developmental milestones. He was preliminarily diagnosed with obsessive-compulsive disorder by his primary care provider and prescribed sertraline 50 milligrams (mg) daily to which the patient was not adherent.

Within two weeks, the patient presented to the emergency department with suicidal ideation in the context of hearing demons. There were no other concerns including homicidal ideation. Aside from a positive urine drug sample for cannabis, the patient did not present with any medical illness precluding a psychiatric diagnosis, and thereby, he was admitted to inpatient psychiatry for seven days (Table 1). During his first hospitalization, the patient revealed that the onset of his repetitive cleaning behavior was prompted by the fear of bringing SARS-CoV-2 home, although he persistently denied a fear of contracting the illness. He scored a six on the Children's Yale-Brown Obsessive-Compulsive Scale (CY-BOCS), but the patient minimally participated in the questionnaire displaying paranoia and guardedness. He also disclosed intrusive thoughts, preference for self-isolation, and detailed food selection methods. The patient was restarted on sertraline 50 mg daily, which was titrated to 75 mg daily prior to discharge; he was also provided hydroxyzine 25 mg three times per day as needed for anxiety. He participated in family, group, and individual therapy. The patient displayed an improvement in his obsessive thoughts, decrease in intrusive thoughts, and lack of handwashing rituals prior to discharge.

After his discharge from his first psychiatric hospitalization, the patient gradually resumed his ritualistic cleaning behavior. He self-isolated and engaged in poor self-care, including not showering or wearing clean clothes. The patient was not distressed with the time spent cleaning, denied intrusive thoughts of cleaning, and noted that cleaning did not provide him joy. He denied auditory hallucinations. The patient did not meet the criteria for major depressive disorder at the time. His sertraline was increased to 100 mg daily within two months of discharge from hospitalization. The patient's cleaning behaviors worsened, including excessive cleaning that led to the destruction of products, running washers without items in the machine, and cooking and eating specific food groups. Although the patient denied difficulty sleeping, there was concern from his parents that he was sleeping minimally. He displayed paranoid and disorganized thinking exacerbated by poor insight and judgment. Risperidone 0.5 mg nightly was added to his medication regimen and increased to 1 mg nightly due to lack of symptom improvement.

For two months, the patient displayed mild improvement in cleaning and cooking behaviors, affect, and self-isolation in the context of persistent lack of personal hygiene and nudity at home. He also disclosed anhedonia. There was ongoing concern for medication nonadherence. At this time, sertraline was titrated from 150 to 200 mg daily, and risperidone was increased to 1.5 mg nightly. The patient reported one month later that he stopped taking risperidone due to insomnia. Despite reporting a positive mood, the patient persistently displayed blunted affect. Sertraline and risperidone were discontinued, and fluoxetine 10 mg daily was started.

After two months on fluoxetine 10 mg, the patient presented with decreased cleaning behavior but still insisted on an unhealthy diet that he personally prepares, spent a minimal amount of time outside of his room, did not wear clothes inside the home, and refused to brush his teeth consistently leading to cavities. He emphasized that he has no fear of germs. The patient was also prescribed olanzapine 2.5 mg daily, but there was persistent concern for medication nonadherence.

Due to worsening symptoms over one month, the patient was admitted to inpatient psychiatry for locking himself in his room due to fear of contamination, not completing his activities of daily living such as showering and eating, refusing to take his medications and interact with others, and struggling with anhedonia including academics for which he failed to move up a grade (Table 1). During his hospitalization, the patient was continued on liquid fluoxetine 10 mg daily as well as hydroxyzine 10 mg three times per day as needed and titrated up to olanzapine 5 mg and additionally received melatonin 3 mg nightly for sleep. Lab work displayed significantly low vitamin D levels at 7 nanograms/milliliter (ng/mL), which was repleted with vitamin D 2,000 units daily. During hospitalization, the patient's affect and communication improved, and he tolerated his medications well without significant side effects supported by an Abnormal Involuntary Movement Scale (AIMS) of zero. He displayed gradual improvement evidenced by decreased obsessions, compulsions, paranoia, and withdrawal from others. He still only ate certain food groups. He participated in family, group, and individual therapy well. Upon discharge, he demonstrated improvement in insight and judgment without symptoms of suicidal or homicidal ideation, psychosis including hallucinations or delusions, or mania. He was discharged with the diagnoses of obsessive-compulsive disorder and delusional disorder, but early-onset psychosis due to schizophrenia must still be ruled out. The patient will continue follow-up in the outpatient setting.

## 3. Discussion

It is well documented that the fear of getting the COVID-19 infection leads to intense experiences such as loneliness, stress, anxiety, fear, anger, depression, paranoia, and even suicide attempts [3, 35]. Moreover, there is literature supporting a relationship between adverse childhood experiences and adulthood OCD [36]. Adverse childhood experiences could include severe illness from SARS-CoV-2 or known deaths from COVID-19, but just fear of COVID-19 may not be applicable [37]. The increased stress of the pandemic, worsened by societal distress, isolation, and widespread fear, is likely to impact both the etiology and maintenance of OCD symptoms [38].

The hypotheses regarding the mechanism of OCD symptom exacerbation during the COVID-19 pandemic emphasize the impact of hand and surface hygiene as well as the constant barrage of media warnings reiterating the threat of the disease [28]. Such a fear of SARS-CoV-2 could incite symptoms of anxiety and depression, which in adolescents, is proposed to be mediated by negative emotional reactivity [39]. Additionally, there is support that experiential avoidance may transform fear of COVID-19 into OCD symptoms due to cognitive errors [37, 39].

Regardless of the potentially increased prevalence or worsening of OCD symptoms, the efficacy of first-line treatment approaches for OCD has not been disproven. Both cognitive-behavioral therapy and selective serotonin reuptake inhibitors (SSRIs) remain heavily evidence-based treatments [40]. In the work by Alkhamees, the adult patient responded well to escitalopram within one month [29]. In the report by Tiuvina et al., the adult patient did not respond to hydroxyzine and mirtazapine but had success with a regimen of fluvoxamine and benzodiazepine [31].

There have also been studies that demonstrate the efficacy of clomipramine, a tricyclic antidepressant, approved for OCD in patients ages 10 and older [30, 41]. In the report by Sejdiu et al., the adolescent patient initially presented with olfactory hallucinations, which turned out to be a symptom of OCD as opposed to psychosis or a neurological process, that resolved with clomipramine [30]. In this report's adolescent patient, his auditory hallucinations may indicate early-onset psychosis as opposed to a symptom of OCD, hence its resolution with olanzapine. Moreover, given the patient's presentation of flat affect, poor eye contact, monotone speech, paranoia, social withdrawal, lack of personal hygiene, and auditory hallucinations, there is reasonable consideration for an underlying early-onset psychosis. OCD patients do not concurrently have schizophrenia at an increased rate compared to the general population whereas 25% of schizophrenic patients also display obsession-compulsion symptoms, and 12% of these patients have OCD diagnoses [42].

Limitations to this case report include a lack of periodic tracking of symptom severity and progression from onset through the course via validated scales and of obtaining inflammatory markers during the patient's hospitalizations [6].

## 4. Conclusion

Given the growing literature of new-onset or exacerbated OCD in child and adolescent populations as well as psychosis in adults during the COVID-19 pandemic, it is reasonable to promote raising awareness of this phenomenon and screening in child and adolescent patients, even those without a psychiatric history. The effects of the COVID-19 pandemic have been primarily reported as medical outcomes; therefore, the myriad of potentially chronic psychosocial consequences is neither apparent nor assessed especially in child and adolescent populations. Due to the conflicting reports of OCD prevalence and worsening during the pandemic as well as limited reports of psychosis in children and adolescents, further research including long-term follow-up or comparison groups with a larger sample size is needed.

## Figures and Tables

**Table 1:** Mental status exam on each admission for the first and second hospitalizations.

	First hospitalization	Second hospitalization
Appearance	Well-groomed.	Well-groomed.
Behavior	Good eye contact. Activity level is appropriate.	Eye contact is mostly avoided.
Motor	No abnormal movements. Normal gait and station.	No abnormal movements and no psychomotor agitation or retardation.
Speech	Normal rate, rhythm, and volume. Well-articulated.	Low rate, tone, and volume. Difficult to hear at times.
Mood	Appropriate.	“When can I get out of here?”
Affect	Restricted but appropriate to content and congruent to mood.	Euthymic, avoidant, and congruent to mood.
Thought process	Linear and logical.	Poverty of content.
Thought content	Reality-based. Denies auditory and visual hallucinations.	Delusion of “contamination is everywhere.” Auditory and visual hallucinations, obsessive ruminations and compulsions, and responding internally is not observed.
Suicidal ideation	Without ideation, plan, or intent. Without self-injurious behaviors.	
Homicidal ideation		
Insight	No insight.	Poor.
Judgment	Poor.	Poor.
Attention/memory	Adequate attention. Intact memory.	Attention is good and concentration is sustained. Intact immediate and short-term memory.
Intelligence	Average for age based upon fund of knowledge, comprehension, and vocabulary.	

## Data Availability

Data sharing is not applicable to this article as no new data was created or analyzed in this study.
